# St. George's Hospital and the Sunday Fund

**Published:** 1912-03-16

**Authors:** 


### ST. GEORGE'S HOSPITAL AND THE SUNDAY FUND.

The discussion at the meeting of the Council of the Hospital Sunday Fund, reported in another column, illustrates the usefulness of the King's Hospital Fund in enforcing rules which are applicable in due degree to all institutions receiving grants from that Fund. The report of the Lord Mayor on the working of St. George's Hospital shows that he has failed to grasp the grounds of the objections raised by some of the constituents of the Sunday Fund to the action of the distribution committee in making a grant to St. George's Hospital when a similar grant had been withheld by the King's Hospital Fund were raised. These objections were based upon the findings of Sir Edward Fry's committee, governing the relations between the hospital and its medical school, in regard to funds received from the public to defray the cost of treating the sick in voluntary hospitals. It was necessary for the King's Fund to lay down definite regulations as to the keeping of the books and accounts of each hospital under the uniform system. Those regulations St. George's Hospital disregarded, although they were strictly enforced upon and followed by all the other hospitals with medical schools which received a grant from the King's Fund. The object the King's Fund had in view was to prevent the payment of any money, given for the relief of the sick poor in hospitals, to the support and upkeep of the medical school. As Sir W. Church pointed out, the whole trouble has arisen from St. George's Hospital, without notice or warning, ceasing to enter in the uniform system of accounts their discretionary fund at all. By this action the whole responsibility for the withholding of the King's Fund grant, and for the discussion and objection raised at the meeting of the constituents of the Sunday Fund, rests upon the authorities of St. George's Hospital.

In order that this may be made perfectly clear, it may be well to state briefly the position which has arisen: The rule of the Fund, as circulated amongst the hospitals under date April 12, 1905, was that from and after January 1, 1906, no grants will be made from the King's Fund to any hospitals which make payments to or on account of their medical schools out of general funds subscribed for the relief of the sick poor. Sir William Church, in reply to Sir William Collins at the council meeting in December 1910 (see page 18 of the fourteenth annual report), made a statement which has been accepted as guaranteeing, that, no grant will be made to any hospital which makes payments to or on account of its medical school out of general funds subscribed for the relief of the sick poor. St. George's Hospital in the year 1909 did wrongly pay £875 out of the general funds to its medical school, and in a subsequent year a further sum, wrongly also. This wrong has never been put right, and until it is put right by the hospital authorities who are responsible for the departure from the rule, St. George's Hospital must by its own act be ineligible for a grant as the rule stands to-day.

It will be noticed from the discussion at the council meeting of the Sunday Fund that several of the members present were not familiar with the facts. Had they been so, the discussion which arose must have been materially shortened. However, all's well that ends well, and it is fortunate that the Sunday Fund Council decided to pass no resolution, but to leave Mr. West, the treasurer of St. George's Hospital, who was present, to bring to the notice of the St. George's Hospital authorities what transpired at the meeting. The Sunday Fund further determined to ask Mr. West to express the Council's view, which was to secure as far as possible unanimity of practice between the King's Fund and the Sunday Fund. The whole matter can be happily determined by St. George's Hospital repaying to the general funds the sums wrongly paid in 1909 and in 1910 out of the general funds to its medical school. In this way—and it is the right way—St. George's at once can place itself in the position to apply for a grant from the King's Hospital Fund and to base its claim for it upon the circumstance that this step has been taken, and that in future the authorities will enter in the unified system of accounts their discretionary fund as they did formerly. In any case, except only so far as the questions directly affect St. George's Hospital, the matter can have little interest in future for the voluntary hospitals of London and for the public which supports them.

